# On Intention and Fluctuations in the Coordination Dynamics of Animate Movement

**DOI:** 10.3390/e28050556

**Published:** 2026-05-15

**Authors:** Amaury Dechaux, Aliza T. Sloan, J. A. Scott Kelso

**Affiliations:** 1 Human Brain and Behavior Laboratory, Center for Complex Systems, Florida Atlantic University, Boca Raton, FL 33431, USA; 2 Intelligent Systems Research Centre, Ulster University, Derry∼Londonderry BT48 7JL, UK; 3 Institute for the Augmented Human, University of Bath, Bath BA2 7AY, UK

**Keywords:** bimanual movements, coordination dynamics, error, fluctuation, intention, Haken–Kelso–Bunz model, human behavior, noise

## Abstract

Many of life’s biggest dilemmas can be summed up as a tension between holding on and letting go. The very language evokes a notion of intentionality which, for the most part, has evaded scientific understanding. How might we even get a window into it? Important insights have come from a seemingly simple task: wiggling one’s fingers to and fro to the beat of a metronome. As the metronome pace increases to some critical frequency, one coordinative pattern becomes unstable and switches spontaneously to another. Such transitions are typically preceded by critical fluctuations, a predicted feature of self-organization in complex, dynamical systems. Here we address the nature and source of these fluctuations, usually assumed to be: (1) random; (2) of external origin; and (3) of fixed magnitude. We performed an experiment in which participants were instructed to oscillate their fingers in either an in-phase or anti-phase pattern in time with a metronome and instructed them to either “hold-on” or “let-go” should they feel the pattern begin to change, yielding a 2 by 2 within-subjects design. We observed that as the metronome frequency was increased from 1.00 to 3.00 Hz, fluctuations in the relative phase between the fingers were significantly altered both by the starting coordinative pattern as well as the participant’s intention to “hold it on” or “let it go”. Specifically, the intention to hold on to the anti-phase pattern delayed the spontaneous transition to in-phase, an effect that was paired with increased fluctuations beyond the critical frequency. These observations were analyzed under the extended Haken–Kelso–Bunz (HKB) model which describes the non-linear stochastic dynamics of the order parameter (relative phase) as a gradient descent on a certain potential. Our analysis, in line with experimental results, suggests that intention transforms the HKB potential not only by stabilizing unstable coordination states but also (paradoxically) by increasing fluctuations around them. Such findings may offer new interpretative light on the relation between intention and fluctuations in the coordination dynamics of living things.

## 1. Introduction

*If my body functions as a pure mechanism according to the Laws of Nature, what is this ‘I’?*

∼Erwin Schrödinger, *What is Life?*, 1944 [1]

### 1.1. A Window into Intention

George Miller used to tell his graduate students, “I can foul up any one of your experiments by an act of will. Now why doesn’t that make “will” more important than the stuff *you’re* studying?” [2]. A founder of cognitive science, Miller insisted that the mind and its willful expressions could and should be explored scientifically. “Will is terribly important; I just don’t have anything worthwhile to say about it.” Not for lack of desire, studying will scientifically is tricky since the “I” that manifests itself through acts of will may appear to violate the very Laws of Nature to which “I” must abide. For instance, the tendency of biological systems toward preserving their order seems to defy the second law of thermodynamics (i.e., entropy, the natural tendency towards disorder) [1]. Despite advances in neuroscience, physiology, and kinematics, “It’s as if we’re coming to understand the puppet and the strings, but we know nothing about the puppeteer. That remains as much a mystery as it has been since classical Greece” [3]. As Newton [4] concluded, new laws yet to be discovered must describe the *purposeful* motion of living creatures. However, such an endeavor requires a way to experimentally manipulate and quantify intention. How do we get a window into Schrödinger’s profound question, “What is this ‘I’?” The idea pursued here is to set up experimental boundary conditions so that a participant might both act “spontaneously” and “intentionally” to allow for a direct comparison of spontaneous behavior (sometimes viewed as “unconscious”) with acts of will (those involving conscious effort). Phase transitions in bimanual coordination ([5,6,7,8], see [9] for review) afford a possible paradigm.

### 1.2. Spontaneous Phase Transitions

Sudden and spontaneous changes in coordination, often referred to as phase transitions, are ubiquitous in complex systems. One canonical example of a phase transition is the coherent light emitted by a laser. When sufficient energy is pumped into an optical material, photons within spontaneously lock in phase forming a coherent beam of light. In this example, the energy pumped into the system acts as a control parameter, which triggers a phase transition once it reaches a critical value. Since phase transitions are marked by sudden, qualitative changes in observable behavior, they are easily identified and are useful for investigating how patterns emerge and shift in complex systems. In particular, such qualitative changes enable the identification of collective variables, or order parameters, that specify coordinative states and whose non-linear dynamics underlie such state changes.

Importantly, such transitions are also found on all scales of living systems [10,11,12], including human coordination. For example, in classic studies of bimanual coordination, participants are instructed to rhythmically move their index fingers in one of two patterns: in-phase or anti-phase (engaging homologous or non-homologous muscle groups, respectively; [5,6,7,13], see [9,14,15,16] for reviews). When people move their fingers back and forth at a rate that feels comfortable, the dynamics of the two patterns appear quite similar. An interesting phenomenon occurs, however, when a person is tasked to progressively increase the frequency at which they move their fingers when they begin in an anti-phase pattern. As the rhythm approaches a particularly challenging pace (termed the critical frequency), the anti-phase pattern becomes increasingly unstable, and it becomes increasingly likely for the fingers to switch to an in-phase pattern [5,13]. Conversely, when participants begin in the in-phase pattern, this pattern remains stable beyond the critical frequency. In addition to exposing the different dynamical characteristics of these two coordinative regimes, this line of research indicates that the transition from anti-phase to in-phase coordination appears to be completely spontaneous—independent of intention. Indeed, participants in these experiments are specifically instructed “do not intervene” should they feel the coordination pattern between their limbs begin to change—in other words, to let it happen.

### 1.3. Instruction, Intention and Bimanual Coordination

Foundational studies of bimanual coordination exposed spontaneous transitions in human behavior, yet non-linear transitions in coordination can also be induced intentionally. For example, one set of experiments probed “intentional switching” [17,18,19,20,21,22,23,24]. While rhythmically moving the limbs at a particular frequency, participants in these studies were instructed to intentionally switch from one pattern to the other. In another line of research, which we might refer to as “intentional staying” [25], participants were instructed to hold onto a particular coordination pattern and to resist the spontaneous anti-phase-to-in-phase transition where applicable. Taken together, this body of research has shown that the dynamics of coordination, in particular the stability (or instability) of movement patterns, is very sensitive to the participant’s intention, which itself can be influenced by experimental instruction. Participants can switch from the more intrinsically stable in-phase to the less stable anti-phase when instructed to do so (a departure from spontaneous behavior under the ’do not intervene’ instruction set), and they can prevent the transition to in-phase coordination when instructed to hold onto the anti-phase pattern [25,26]. Furthermore, subjects can switch intentionally to the anti-phase pattern beyond the critical frequency. From this, the authors concluded that when a participant intentionally switches, they are not simply selecting from among the available intrinsic patterns; they are actually changing the characteristics of the pattern dynamics. In other words, the intention to switch can act to destabilize one pattern and stabilize the other.

Whereas the foregoing studies demonstrated that coordination dynamics are sensitive to intention, they simultaneously found that intention is constrained by intrinsic dynamics. For example, the transition from in-phase to anti-phase takes longer [17,18,20,22] and appears more cognitively intensive [22,27] than its in-phase-to-anti-phase counterpart, again revealing an inherent stability difference between these two basic patterns. The in-phase pattern remains more stable than its anti-phase counterpart, regardless of whether a participant is instructed to stabilize it [25]. Furthermore, below the critical frequency, the intention to maintain a particular coordination pattern does not appear to influence its pattern stability. Additionally, although participants can prevent the transition to in-phase when instructed to hold onto anti-phase, the anti-phase pattern becomes increasingly unstable as measured by the increasing magnitude of fluctuations in relative phase (a measure of coordination). There is no return to stability as in the cases of spontaneous or intentional switching [6]. In sum, it appears that it is the interaction between intentions and spontaneous dynamics that ultimately determines the system’s trajectory through space and time as well as the fate of its various order parameters [11,28].

### 1.4. Current Study: On Intention and Fluctuations

To reiterate, when intention is at odds with spontaneous dynamics (such as when a participant is told to hold onto anti-phase at rates higher than the critical frequency), fluctuations intensify. We might then say that intention is hidden in (and revealed by) fluctuations [9]. As spontaneous coordination dynamics always guide the system toward stability, behavior that deviates from this flow toward stability is what we often describe as ’intentional’. But what is the nature of these fluctuations and what can fluctuations tell us about the nature of intention?

Here, we compared two modes of bimanual coordination (in-phase and anti-phase) under two instructional sets (“Let go” and “Hold on”). Assuming that the classic anti-phase to in-phase transition does indeed occur spontaneously under the instructions “Do not intervene”, the bimanual paradigm then offers a unique opportunity to probe the nature of intention by now instructing the participants, “Do intervene!” (see also [25]). Changing this boundary condition allows us to compare a situation where behavior unfolds spontaneously (i.e., “as a pure mechanism”) to a situation where intention shapes and transforms collective behavior. Crucially, as the collective behavior of the fingers is described by the Haken–Kelso–Bunz (HKB) model, the way intention transforms the coordination dynamics can be explored by investigating how the HKB model must be modified to accommodate the intentional effects of holding on. Critically, these comparisons must be made within subjects since each person’s intrinsic dynamics are unique, unknown a priori, and must be identified [29,30]. To be perfectly clear, our goal is to first establish empirically that holding on can delay or prevent transitions and has effects on coordinative fluctuations. Then, theoretically, we develop a modified version of the HKB model’s collective (relative phase) dynamics that reproduces our experimental findings. An interesting consequence of this modified HKB model is that intention and fluctuation appear fundamentally interconnected.

## 2. Materials and Methods

### 2.1. Data Collection and Experimental Design

**Figure 1:**
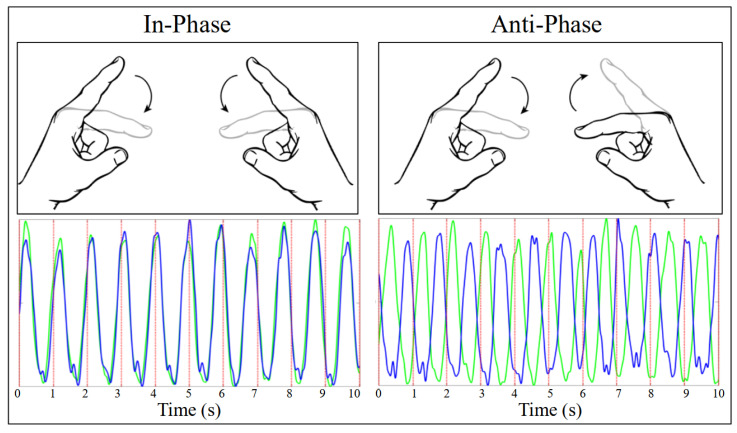
A visual depiction of the in-phase (homologous muscle group, (**left**)) and anti-phase (non-homologous muscle group, (**right**)) coordination patterns and an example of finger movement data (amplitude versus time) corresponding to each of them. For the two graphs, the blue and green curves respectively correspond to the movement of the left and right index finger, whereas the timing of the metronome pulses which the participant is following are shown by vertical red lines (the metronome frequency is 1.00 Hz in this example). These time series were sampled from one participant’s Let-go In-phase and Let-go Anti-phase trials. In-phase and anti-phase are the two intrinsically stable (i.e., without specific training) modes of coordination between two oscillating limbs.

Ten participants (9 right-handed and 1 left-handed as per self-report) were instructed to move their two index fingers back and forth to the beat of a metronome whose frequency progressively increased by 0.25 Hz every 16 cycles starting at 1.00 Hz and peaking at 3.00 Hz by the end of each trial (the first metronome plateau, at 1.00 Hz, lasted 24 cycles). Participants were instructed to move their fingers in one of two coordinative patterns: in-phase, where the two fingers oscillate symmetrically, and anti-phase, where the fingers oscillate in parallel (see Figure 1).

The participants performed these two tasks (i.e., in-phase and anti-phase coordination) under two conditions. The Let-go condition reproduced the “Do not intervene” instructions given in Kelso’s original experiment [5,13]. Specifically, the participants were instructed to let the coordination pattern between their fingers change should they feel that start to happen. In contrast, in the hold-on condition, participants were instructed to try their best to maintain the instructed coordination pattern throughout the trial.

This yielded a 2 × 2 experimental design with four conditions (Let-Go In-Phase, Let-Go Anti-phase, Hold-on In-phase and Hold-on Anti-phase), which all participants performed in this specific order: Let-go In-phase, Let-go Anti-phase, Hold-on In-phase and Hold-on Anti-phase. Although randomizing trial order is a standard strategy for minimizing order effects, we found that trial order had a very large effect on participant behavior during preliminary testing. Specifically, participants who were instructed to hold on before they were instructed to let go found it extremely difficult to let go when the time came. In fact, none of these participants exhibited clean switches from anti-phase to in-phase, and so no further analysis could be performed regarding super-critical stability—the topic of our study. We, therefore, decided to design our protocol around this limitation by fixing the trial order. At the same time, we worked to minimize effects of fatigue and practice by keeping trial lengths short (about 85 s each), offering breaks between each trial, and prefacing the experimental trials with sufficient practice such that all participants were achieving reasonable performance under both coordinative patterns. While we acknowledge that our approach can only reduce rather than eliminate order effects, the results of our preliminary study strongly suggest that randomizing the trial order would have yielded much larger order effects.

**Figure 2:**
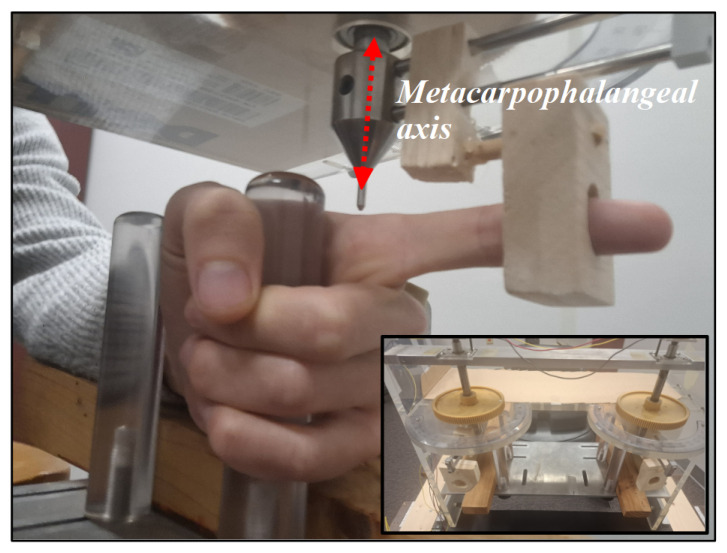
The experimental apparatus. The motion of the participant’s two index fingers was restricted to a rotation in the horizontal plane about the metacarpophalangeal joints whose axis is shown by a dashed red line.

To stabilize the participant while they performed the coordination tasks, their two index fingers were inserted in an apparatus consisting of two freely rotating supports. These supports were designed to allow flexion and extension of the index fingers about the metacarpophalangeal joint in the horizontal plane (see Figure 2). A potentiometer mounted over the axis of rotation of each finger provided an analog signal which was digitized and recorded at a rate of 200 Hz. To synchronize the finger motion time series with the metronome, the same converter was used to sample the signal provided by a microphone recording the metronome pulses. Each trial thus provided us with three time-locked series of about 85 s starting 2 s before the first metronome pulse and ending 2 s after the last. These time series were subsequently imported to Matlab (R2025a) for analysis.

### 2.2. Data Processing and Estimation of the Relative Phase

**Figure 3:**
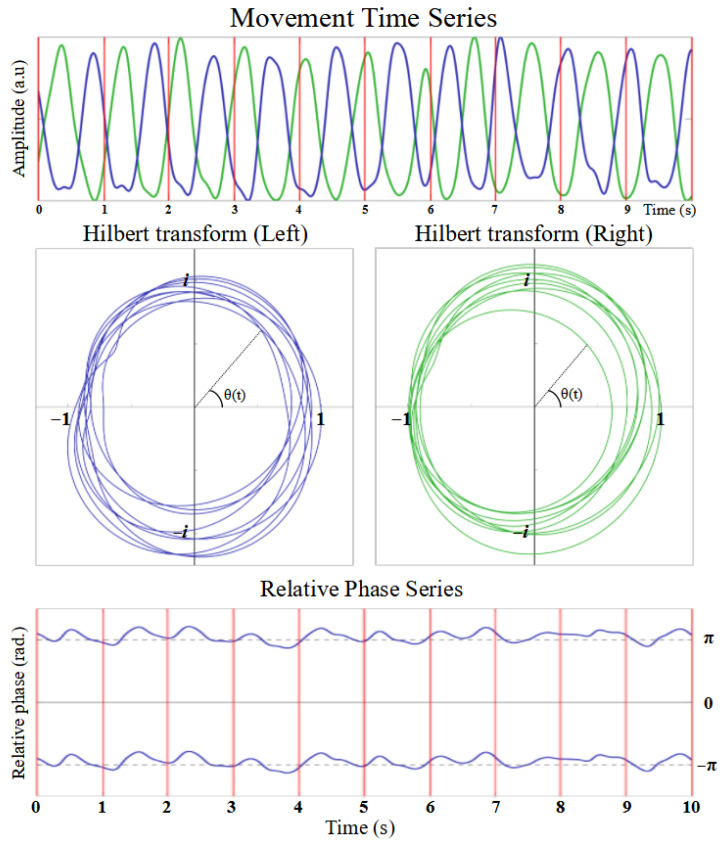
A point-wise estimate of the relative phase between the two index fingers (left in blue and right in green) is obtained according to the method described in [31]. The movement data are first filtered and normalized (**top**). A Hilbert transform is then applied to each time series (**center**) to obtain a estimate of the phase for each finger. The relative phase (**bottom**) is obtained by subtracting the phase of the right dominant hand (in this example) from the left. In the top panel, the movement of the right and left index fingers are shown in green and blue respectively while the vertical red lines mark the metronome pulses (1 every second in this example). The result of the application of the Hilbert transform to the movement data is shown in the complex plane in the center panels. The Hilbert transform of a real signal (x(t)∈R) yields a complex signal (y(t)∈C) whose argument θ(t) (in radians) can be used as an estimate of the phase of the original signal. Subtracting the estimate of the phase obtained for one finger from the estimate of the phase obtained for the other yields a point-wise estimate of the relative phase between the fingers (in radians) shown by the blue curve in the bottom panel. Vertical red lines mark the pulses of the metronome, whereas the solid and dashed horizontal lines denote the values of the relative phase corresponding to in-phase coordination (0+2kπ, k∈Z radians) and anti-phase coordination (π+2kπ, k∈Z radians) between the fingers. As can be visually observed from the movement data (**top**) and read from the value of the relative phase (**bottom**), the participant is coordinating their fingers in the anti-phase pattern. This sample was taken from the first metronome plateau of the Let-go Anti-phase trial.

We began processing the timeseries data by extracting the metronome pulses from the audio signal by thresholding its envelope. Each of the finger time series was first numerically filtered (forward and backward in zero-lag fashion) using a 4th order low pass Butterworth with a cutoff frequency of 10 Hz. We then obtained estimates for the instantaneous phase ($\theta_{R}\left(t\right)$, $\theta_{L}\left(t\right)$) at each time point, *t*, using a Hilbert transform [31] performed independently over each metronome plateau for normalization purposes. The relative phase between the fingers was computed by subtracting the non-dominant hand’s phase from that of the dominant hand (see Figure 3). Finally, we estimated the cycling frequency of each finger ($f_{R}\left(t\right)$, $f_{L}\left(t\right)$) by the ridge of a scalogram (i.e., the highest power frequency as an approximation of the instantaneous frequency) obtained through a wavelet transform [32].

## 3. Results

Two participants were excluded from the analysis, one for failing to follow the metronome with reasonable precision and another for generally failing to generate a sufficiently clear signal at medium to high metronome frequencies. The following analysis focuses on the remaining eight participants.

### 3.1. Cycling Frequency of the Fingers

We first assessed how well participants kept up with the metronome by quantifying the deviation of each participant’s movement frequency from that of the metronome. To this end, we took the average of each index finger’s approximate instantaneous frequency over the last 8 cycles of each metronome plateau (where we assume that the relative phase has reached a steady state) and computed the deviation (*s*) as

(1) $$s=\frac{{\bar{f}}_{i}}{f_{m}}-1$$

where $f_{m}$ is the metronome frequency for a given plateau and ${\bar{f}}_{i}$ the average movement frequency in the last 8 cycles of that plateau. The frequency of the metronome was significantly different from the frequencies of the dominant hand (*M* = 0.053, *SD* = 0.10; *t*(287) = 8.96; *p* < $10^{-5}$) and the non-dominant hand (*M* = 0.053, *SD* = 0.10; *t*(287) = 9.28; *p* < $10^{-5}$). Participants paced themselves slightly faster (about 5%) than the metronome on average across all plateaus and conditions. A two-way ANOVA with factors of intention (“Let-go” or “Hold-on”) and metronome frequency performed for each hand revealed a significant main effect of metronome frequency for the dominant hand [*F*(8, 270) = 1.96; $\eta^{2}$ = 0.05; *p* = 0.05] and a nearly significant main effect of metronome frequency for the non-dominant hand [*F*(8, 270) = 1.85; $\eta^{2}$ = 0.05; *p* = 0.06]. However, the effects of metronome frequency were largely outweighed by the participant’s intention when considering the cycling frequency of both the dominant hand [*F*(8, 270) = 7.60; $\eta^{2}$ = 0.03; *p* = $6\times 10^{-3}$] and the non-dominant hand [*F*(8, 270) = 11.35; $\eta^{2}$ = 0.04; *p* < $10^{-3}$], with no significant interactions. Overall, participants consistently moved their fingers faster than the metronome by about 3.5% when instructed to let go and by about twice that amount, 7.1%, when instructed to hold on (a sign, perhaps, of increased intentional effort). The average deviation was largest for the slowest metronome paces, starting at about 7.1% in the 1.00 Hz plateau and steadily decreasing over the course of each trial to about 3% in the 3.00 Hz plateau. Next, we compared the cycling frequency of each participant’s finger movements. Quantifying the deviations between each participant’s index fingers using a method analogous to Equation (1) revealed that the fingers oscillated at the same frequency, statistically speaking (*M* < $10^{-3}$, *SD*: 0.02; *t*(287) = 0.53; *p* = 0.60), and that there were no main effects of intention [*F*(1, 270) = 2.31; $\eta^{2}$ = $8\times 10^{-3}$; *p* > 0.10] nor of metronome frequency [*F*(8, 270) = 2.31; $\eta^{2}$ = 0.03; *p* = 0.40]. In sum, participants followed task instructions reasonably well.

### 3.2. Patterns of Coordination Between the Fingers

Having confirmed that the participants paced their fingers as instructed, we next characterized the patterns of coordination between the fingers, as quantified by relative phase.

#### 3.2.1. Relative Phase Dynamics and Phase Transitions

**Figure 4:**
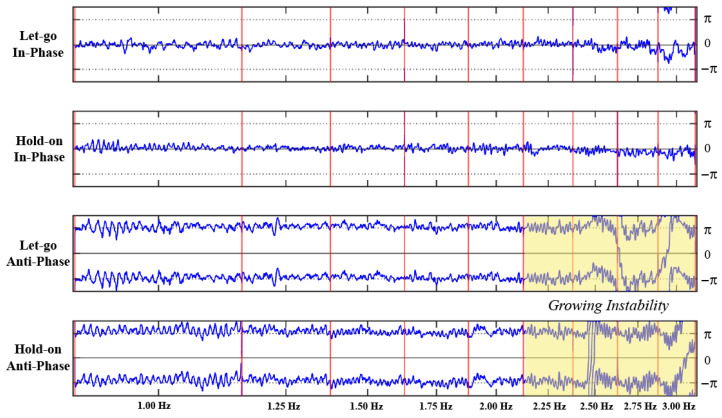
The relative phase (blue time series, in radians) between a participant’s finger movements as a function of time in each of the four trials of the experiment (from top to bottom, Let-go In-phase, Hold-on In-phase, Let-go Anti-phase and Hold-on Anti-phase). In each of the four graphs, the vertical red lines delimit the beginning and end of each of the 9 metronome plateaus whose frequency is denoted at the bottom. As can be observed in the third row, this participant did not spontaneously transition from anti-phase to in-phase during the Let-go Anti-phase trial. At the same time, however, one can observe the large difference in stability between the two coordination patterns (compare the 1st and 2nd with 3rd and 4th rows), where performing an anti-phase pattern yields large fluctuations around ϕ=±π compared to the small fluctuations around ϕ=0 when the participant is performing the in-phase pattern. Note also the irregularity at higher frequencies, circa 2.50 Hz onward, when the participant is performing the anti-phase pattern.

**Figure 5:**
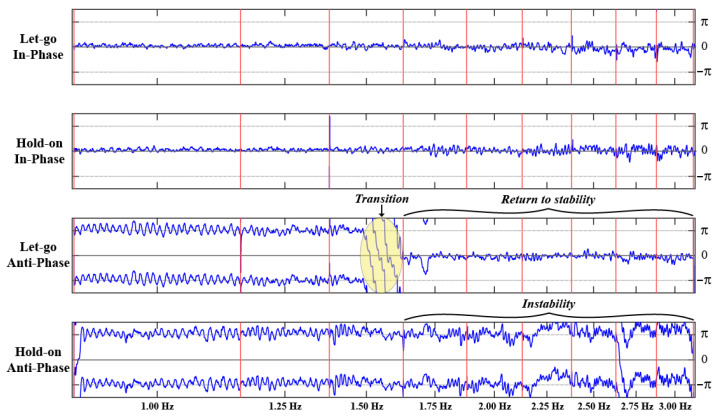
The relative phase (blue time series, in radians) between a participant’s finger movements as a function of time in each of the four experimental trials (from top to bottom, Let-go In-phase, Hold-on In-phase, Let-go Anti-phase and Hold-on Anti-phase). In each of the four graphs, the vertical red lines delimit the beginning and end of each of the 9 metronome plateaus whose frequency is denoted at the bottom. A transition from anti-phase to in-phase (ϕ=±π to ϕ=0) can be observed in the second half of the third metronome plateau of the Let-go Anti-phase trial (3rd row). Conversely, after being instructed to hold on to the anti-phase pattern (4th row), the participant is able to prevent this transition, but with increasing instability.

**Figure 6:**
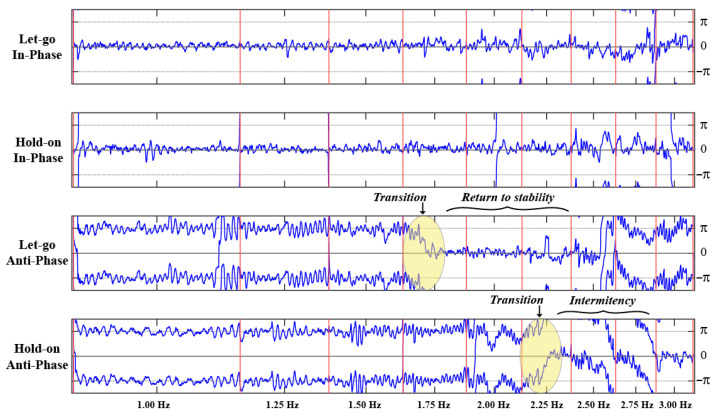
The relative phase (blue time series, in radians) between a participant’s finger movements as a function of time in each of the four experimental trials (from top to bottom, Let-go In-phase, Hold-on In-phase, Let-go Anti-phase and Hold-on Anti-phase). In each of the four graphs, the vertical red lines delimit the beginning and end of each of the 9 metronome plateaus whose frequency is denoted at the bottom. A transition from anti-phase to in-phase (ϕ=±π to ϕ=0) can be observed at 1.75 Hz in the Let-go Anti-phase trial (3rd row). In contrast to the data shown in Figure 5, this participant was not able to prevent the transition from anti-phase to in-phase by holding on but delayed it to 2.25 Hz (4th row).

Examining the dynamics of relative phase in each of the four experimental conditions revealed three characteristic behaviors: no transition, prevented transition, and delayed transition. Three participants did not spontaneously transition from anti-phase to in-phase at any point during the Let-go Anti-phase and Hold-on Anti-phase trials (an example is shown in Figure 4). The remaining five participants transitioned from anti-phase to in-phase somewhere between 1.50 Hz and 2.25 Hz when instructed to let go. Three of the participants who switched in the Let-go condition were able to prevent a switch to the in-phase pattern when instructed to hold on to the anti-phase pattern (an example is shown in Figure 5). In contrast, the other two participants only managed to delay that transition by holding on from 1.75 Hz to 2.25 Hz and from 1.50 Hz to 2.50 Hz in the Let-go Anti-phase and Hold-on Anti-phase trials, respectively (an example is shown in Figure 6).

This preliminary inspection illustrates two key results regarding a participant’s intention to hold on to or let go of a particular pattern. First, comparing the relative phase dynamics in the Let-go In-phase and Hold-on In-phase trials (first and second row of Figure 4, Figure 5 and Figure 6), the intention to stabilize the in-phase coordination pattern does not appear to have any effect. Second, while holding on to an anti-phase pattern allows a participant to prevent or delay a transition to in-phase, holding on to this coordination pattern at super-critical frequencies comes at a cost in the form of large fluctuations in the relative phase (in comparison to the return to stability characterizing a spontaneous transition to in-phase). This is particularly visible in Figure 5, comparing the “noisiness” of the relative phase pre- and post-transition in the Let-go Anti-phase trial (e.g., the 1.25 Hz versus 2.00 Hz plateau) versus the large fluctuations around ±π when the participant is maintaining an anti-phase pattern in the last few metronome plateaus of the Hold-on Anti-phase trial (e.g., 2.50 Hz and above).

#### 3.2.2. Pattern Stability and Fluctuations in Relative Phase

To determine quantitatively whether maintaining the anti-phase pattern does indeed come with increased fluctuations, we computed the circular mean (μ) and standard deviation (σ) of the relative phase over the last 8 cycles (last half) of each metronome plateau, where we assume that the relative phase has reached a steady state. These quantities are given by

(2) $$\begin{array}{cc} Z_{1} & =\frac{1}{N}\sum_{\tau }e^{i\phi [\tau ]}\\ \mu & =\arg(Z_{1})\\ \sigma & =\left(-2\log(\right|Z_{1}{\left|)\right)}^{1/2} \end{array}$$

Given that some participants reported having difficulty “letting-go” in the Let-go trials, it is important to study only those who spontaneously transition from anti-phase to in-phase in the Let-go Anti-phase trial. The reason is that only the presence of a phase transition in the Let-go Anti-phase trial guarantees that a participant is not holding on when the instruction is to let go. To enable a fair assessment of possible intentional effects, we thus focused our analysis on those data that allow a clear comparison of holding on and letting go. To account for the fact that participants switch at different frequencies (i.e., intrinsic dynamics are specific to the individual), we split our measures of circular mean and standard deviation into three groups based on metronome frequency. Having visually established the metronome plateaus at which participants transitioned, we defined low, medium, and high frequency ranges corresponding to the first three (1.00 to 1.50 Hz), middle three (1.75 to 2.25 Hz), and last three (2.50 to 3.00 Hz) metronome plateaus. Splitting the data in this way allowed us to compare the magnitude of fluctuations across participants in frequency regions generally below the transition frequency (i.e., low frequency region), near the transition frequency (i.e., medium frequency region), and above the transition frequency (i.e., high frequency region).

To test whether fluctuations were greater in the Hold-on Anti-phase trial at high metronome frequencies, we performed a three-way ANOVA, taking intention, starting pattern, and metronome frequency region as factors on the standard deviation of the relative phase. The ANOVA revealed significant main effects of the metronome frequency region [F(2,168)=10.19; $\eta^{2}=0.12$; $p<10^{-5}$] and the starting pattern [F(1,168)=15.47; $\eta^{2}=0.09$; $p<10^{-4}$] and no significant main effect of intention [$F\left(1,168\right)=5\times 10^{-4}$; $\eta^{2}<10^{-5}$; p=0.98], as expected. The ANOVA also revealed a marginally significant interaction between the three factors [F(2,168)=2.89; $\eta^{2}=0.01$; p=0.057], while the only significant pairwise interaction was between intention and metronome frequency region [F(1,168)=3.25; $\eta^{2}=0.03$; p=0.04].

This suggests, in line with previous research, that the effect of intention to hold on or let go is highly dependent on the specific pattern the participant is instructed to maintain as well as the frequency at which they are paced when doing so. Since it is known that holding on to the intrinsically stable in-phase pattern does not generally change the relative phase dynamics, we performed this analysis again, this time splitting the data according to the starting pattern. As expected, considering only the trials in which the participants started in the in-phase pattern (i.e., Let-go In-phase and Hold-on In-phase), we found no main effect of intention [F(1,84)=0.13; $\eta^{2}=0.01$; p=0.71] and no interaction between intention and metronome frequency region [F(2,84)=0.001; $\eta^{2}<10^{-3}$; p=0.99] on the standard deviation of the relative phase. The only significant effect in these trials was the main effect of the metronome frequency region [F(2,84)=18.02; $\eta^{2}=0.42$; $p<10^{-6}$].

The picture is very different for trials in which the participants started in the anti-phase pattern (i.e., Let-go Anti-phase and Hold-on Anti-phase). Here, the only significant effect was the interaction between intention and metronome frequency region [F(2,84)=4.17; $\eta^{2}=0.10$; p=0.02]. By themselves, neither intention [F(1,84)=0.04; $\eta^{2}<10^{-3}$; p=0.84] nor metronome frequency region [F(2,84)=1.57; $\eta^{2}=0.03$; p=0.21] were significant as main effects. In sum, these analyses confirm what we observed by visually inspecting our data, namely that fluctuations were greater in the Hold-on Anti-phase trial at high metronome frequencies. Table 1 summarizes the average means and standard deviations of the relative phase under the three metronome frequency regions. Note the elevated standard deviation in the Hold-on Anti-phase trial at high metronome frequencies (i.e., generally super-critical frequencies) relative to other conditions and metronome settings.

### 3.3. Model-Based Analysis

To investigate the source of these super-critical fluctuations, we turned to the Haken–Kelso–Bunz model (HKB; [8,33,34]). It has been shown that the order parameter dynamics (here, the dynamics of the relative phase) are well-described by the HKB model, a model whose predictions were subsequently verified experimentally in great detail (see [35] for review). However, part of the difficulty in characterizing the source of our experimentally observed fluctuations comes from the fact that, according to this model, the standard deviation of the relative phase (i.e., variability) is determined by two competing effects: the amount of noise within the system whose random “pushes” produce fluctuations around its equilibrium points (i.e., in-phase and anti-phase), and the dynamical stability of these equilibrium points which tends to “pull” these coordinative states back toward stability. The relative importance of these two effects is reflected by the standard deviation, which grows with the amount of noise and shrinks with the dynamical stability.

Since it is known that the dynamical stability of the in-phase and anti-phase coordination patterns decreases as the cycling frequency increases (and the HKB potential shallows), explaining the large standard deviation when participants are holding on to the anti-phase pattern at super-critical frequencies thus requires us to distinguish between two competing hypotheses. The first is simply that the level of noise somehow increases due to the participant’s intention to hold on, whereas the second points to the continued loss of stability as the participants cycle their fingers at increasingly fast paces (while the noise level remains constant).

In the following sections, we present a basic form of the HKB model (Section 3.3.1), an extended form of HKB which includes a term representing a participant’s intention to stabilize a given coordination pattern (Section 3.3.2), relate parameters of the extended HKB model to experimentally measured quantities (i.e., the mean and SD of relative phase) (Section 3.3.3), and finally use those model parameters to explore plausible sources of super-critical fluctuations (Section 3.3.4). Within this extended HKB model, our investigation will show that explaining the experimental facts requires that the participant’s intention to hold on transforms the collective dynamics such that it stabilizes the coordinative pattern (i.e., anti-phase) and, at the same time, increases the level of noise (which thus can no longer be assumed constant [5,33]).

#### 3.3.1. The Haken–Kelso–Bunz Model

**Figure 7:**
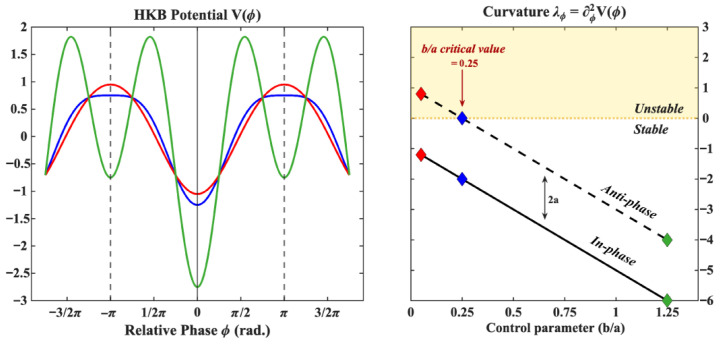
The Haken–Kelso–Bunz (HKB; [8,33]) model describes the dynamics of the relative phase between two oscillating components as a gradient descent on a potential V(ϕ) (shown on the left for three different parameter values) whose shape depends on two parameters a,b>0. The ratio b/a determines the number of local minima which correspond to attractor states for the relative phase. For b/a>0.25, the HKB model predicts the existence of two stable fixed points located at 0 (in-phase coordination) and π (anti-phase coordination) (green curve where b/a=1.25). At b/a=0.25 (blue curve) the anti-phase stable fixed point undergoes a pitchfork bifurcation and becomes (locally) neutrally stable. Below this, the anti-phase coordination patterns becomes a repeller (red curve where b/a=0.05). The stability of a fixed point can be indexed by the curvature of the potential in its vicinity, shown on the right for the in-phase (solid line) and anti-phase (dashed line) fixed points as a function of the control parameter (b/a). In one dimension, stable and unstable fixed points are characterized by a negative and positive curvature respectively, a large negative curvature reflecting a more stable fixed point. As observed in the panel on the right, the HKB model predicts that the anti-phase pattern is intrinsically less stable than the in-phase pattern (the difference being given by Δ=2a) no matter the value of the control parameter (i.e., at every oscillation period). The color of each diamond indicates control parameter values corresponding to the potentials shown on the left.

The Haken–Kelso–Bunz model (HKB; [8,33,35]) describes the stochastic non-linear dynamics of the relative phase between two oscillators (e.g., two limbs) as a gradient descent on a potential V(ϕ) whose shape depends on two positive parameters *a* and *b* (see Figure 7):

(3) $$\begin{array}{cc} \dot{\phi } & =-\frac{d}{d\phi }V\left(\phi \right)+\sqrt{Q}\xi_{t}\\ V(\phi ) & =-a\cos(\phi )-b\cos(2\phi ) \end{array}$$

where ϕ is the relative phase ($\dot{\phi }$ being its rate of change) and $\xi_{t}$ is a delta-correlated gaussian noise whose magnitude is indexed by a parameter *Q*. The ratio b/a, a control parameter, is proportional to the oscillation period. For b/a>0.25 (i.e., low pacing frequency), the model predicts the existence of two stable fixed points located at 0 rad (in-phase coordination) and π rad (anti-phase coordination). Moving towards b/a≤0.25 (i.e., from low to high pace), the anti-phase stable fixed point undergoes a pitchfork bifurcation and becomes unstable, resulting in the experimentally observed “phase transition” from anti-phase to in-phase. The stability of the two coordination patterns is indexed by the curvature of the potential in their vicinity. This curvature, which is a negative number for a stable fixed point, is given by

(4) $$\begin{array}{cc} \lambda_{0} & =\frac{d^{2}}{d\phi^{2}}V\left(0\right)=-4b-a\\ \lambda_{\pi } & =\frac{d^{2}}{d\phi^{2}}V\left(\pi \right)=-4b+a \end{array}$$

**Figure 8:**
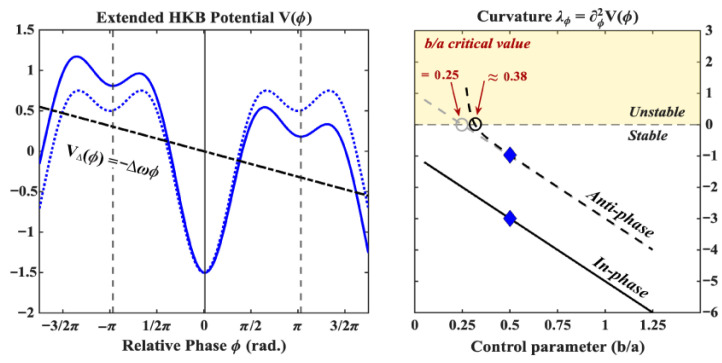
Introducing a detuning parameter Δω [34,36] modifies the potential landscape as shown on the left where the resulting potential (solid blue curve) is the sum of the original HKB potential (dashed blue curve, drawn here for b/a=0.50) and the detuning curve $V_{\Delta }\left(\phi \right)=-\Delta \omega \phi$ (dashed black line, with Δω=0.1). The effect on the dynamics is twofold: the fixed points of the system (vertical lines, left panel) are shifted and their stability, as indexed by the curvature of the resulting potential shown on the right (blue diamonds), is modified in a non-linear fashion close to instability points. The critical value of the control parameter for which the anti-phase pattern becomes unstable is shifted to the right, meaning one can expect transitions at lower frequencies depending on the value of the detuning parameter.

Given that *a* and *b* are positive, the model predicts that the in-phase pattern is intrinsically more stable than its anti-phase counterpart, with the difference in stability between the two fixed points given by Δ=2a. Numerous extensions and variants of this model have been proposed over the years, expanding the model’s explanatory power to a wide range of phenomena (not necessarily pertaining to inter-limb coordination, see e.g., [35] for a review). One particular extension, introduced in [34] and applied to inter-limb coordination in [36], takes into account the fact that the two coordinating limbs may not have the exact same dynamical properties (as it was assumed in the original model) and, in particular, allows one to model the fact that these limbs may not share the same intrinsic (eigen) frequency (when modeled as non-linear oscillators). This can be controlled for simply by adding a linear symmetry breaking term to the potential such that the model becomes

(5) $$\begin{array}{cc} \dot{\phi } & =-\frac{d}{d\phi }V\left(\phi \right)+\sqrt{Q}\xi_{t}\\ V(\phi ) & =-\Delta \omega \;\phi -a\cos(\phi )-b\cos(2\phi ) \end{array}$$

where $\Delta \omega =\frac{\omega_{1}^{2}-\omega_{2}^{2}}{\Omega }$ is the *detuning* parameter representing the tendencies of the limbs to oscillate at their individual preferred frequencies ($\omega_{1}$ and $\omega_{2}$) and whose magnitude is inversely proportional to the rate Ω (the oscillating frequency of the coupled system). When the difference in preferred frequency is small, one can use the approximation $\Delta \omega \approx w_{1}-w_{2}$. The notable effect of this term on the dynamics of the relative phase is to shift the fixed points away from their canonical values, $\phi^{*}=0$ and $\phi^{*}=\pm \pi$, which affects their dynamical stability in a non-linear fashion, in particular close to instability points (see Figure 8). While this term is generally encountered in the context of coordination between limbs with vastly different dynamical properties (e.g., an arm and a leg [36]) or between the coordination of sensory stimuli and finger movements [34], a previous study on the effects of intention on the relative phase dynamics showed that, when participants are instructed to *intentionally switch* between the in-phase and anti-phase coordination patterns, they do so preferentially using their non-dominant hand [21]. This may be interpreted as evidence that intentionality increases the natural symmetry breaking effect of handedness.

#### 3.3.2. Introducing Intentionality into HKB

**Figure 9:**
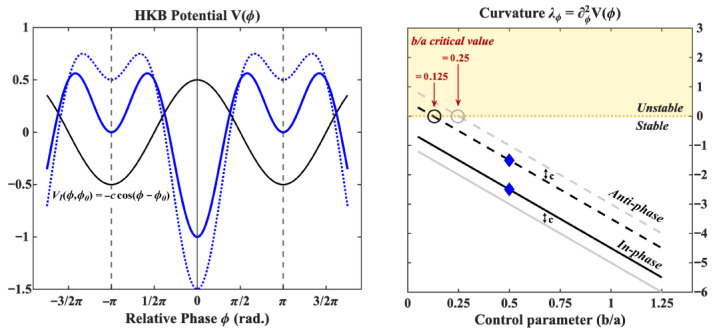
Introducing intentional forcing modifies the potential landscape of the dynamic as shown on the left where the resulting potential (solid blue curve) is the sum of the original HKB potential (dotted blue drawn here for b/a=0.50) and the forcing curve $V\left(\phi ,\phi_{0}\right)=-c\cos\left(\phi -\phi_{0}\right)$ (solid black with c=0.5 and $\phi_{0}=\pi$). This term acts on the two fixed points differently depending on which of them is being intentionally forced (as determined by $\phi_{0}$). As shown on the right for $\phi_{0}=\pi$ (the participant is intentionally forcing an anti-phase coordination), it increases the stability of the fixed point by *c* (the curvature becomes more negative) and decreases the stability of the other. The critical value of the control parameter for which the anti-phase pattern becomes unstable is either shifted to the left to b/a=0.25(1−c) when this pattern is being held onto and to the right to b/a=0.25(1+c) when the in-phase pattern is being held onto. In this example, b/a is shifted leftward to 0.125.

Introducing intentionality into HKB amounts to finding an appropriate perturbation to the potential landscape V(ϕ) such that the intentionally forced dynamics match experimental results (e.g., [18,25,28,37]). A very basic requirement comes from the fact that participants in inter-limb coordination studies are perfectly capable of intentionally switching from one coordination pattern to the other when instructed to do so. The intentional term should thus, at minimum, be able to *destabilize* a particular coordination pattern such that a transition may occur toward the other pattern irrespective of the coordination pattern they are switching from (i.e., transitioning from in-phase to anti-phase should be allowed) and independently of the limbs’ oscillation frequencies. A second requirement, exemplified by the results of our study (see also [28]), stems from the fact that participants are capable of intentionally preventing or delaying a spontaneous phase transition from anti-phase to in-phase when approaching the critical frequency. The intentional term should thus also be able to *stabilize* the anti-phase pattern, allowing for its continued existence at higher pacing frequencies in comparison to the “non-intentional case” (note that only destabilizing the in-phase pattern is not enough here). Finally, studies have repeatedly shown that intentionality *interacts with* but crucially *does not supersede* the intrinsic dynamics prescribed by the HKB model. In the context of our study on the intentional stabilization of a given coordination pattern, this implies that it is more difficult to stabilize the intrinsically less stable anti-phase pattern than its in-phase counterpart (larger standard deviation when holding on, see Table 1 but also [25]). The same conclusion is reached when the participant is asked to transition from one pattern to the other. Transitioning from the intrinsically less stable anti-phase mode to the intrinsically more stable in-phase mode is significantly faster (see e.g., [18]) and less cognitively demanding [22,27] than the reverse transition from in-phase to anti-phase. This important fact thus points us towards choices of intentional terms that interact with the original dynamics but otherwise leave them unchanged. In [37], Schöner and Kelso propose a minimal additive perturbation of the potential landscape that satisfies these requirements. The model is modified such that

(6) $$\begin{array}{cc} V(\phi ) & =-\Delta \omega \;\phi -a\cos\left(\phi \right)-b\cos\left(2\phi \right)+V_{I}\left(\phi ,\phi_{0}\right)\\ V_{I}\left(\phi ,\phi_{0}\right) & =-c\cos(\phi -\phi_{0}) \end{array}$$

where $\phi_{0}$ is the coordination pattern that a participant is instructed to intentionally stabilize (or switch to) and c≥0 a parameter measuring the “strength” of intention in relation to the intrinsic dynamics. It is easy to see how this *intentional forcing* term fulfills the three requirements we put forth. Depending on the value of $\phi_{0}$, it both increases the stability of a given mode of coordination (anti-phase when $\phi_{0}=\pi$ and in-phase when $\phi_{0}=0$) and destabilizes the other. The stability of the system’s fixed points (assuming Δω=0) is given by

(7) $$\begin{array}{cc} \lambda_{0} & =\frac{d^{2}}{d\phi^{2}}V\left(0\right)=-4b-a\pm c\\ \lambda_{\pi } & =\frac{d^{2}}{d\phi^{2}}V\left(\pi \right)=-4b+a\pm c \end{array}$$

where *c* counts negatively for the pattern being stabilized (increasing its stability) and negatively for the other (decreasing its stability, see Figure 9). Provided that *c* is roughly commensurate with *a* and *b*, the original dynamics also remain an important factor in the relative phase dynamics. In this form, intentionality thus only interacts with, but does not supersede, the intrinsic dynamics prescribed by the HKB model.

**Figure 10:**
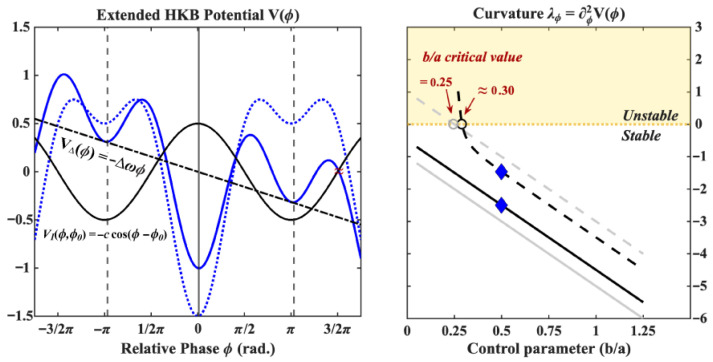
The extended HKB model including both detuning ($V_{\Delta }\left(\phi \right)=-\Delta \omega \phi$) and intentional forcing ($V_{I}\left(\phi ,\phi_{0}\right)=c\cos\left(\phi -\phi_{0}\right)$) terms transforms the original HKB potential into the extended HKB potential as shown on the left by the dotted and solid blue curves respectively, where the anti-phase pattern is intentionally forced ($\phi_{0}=\pi$, the other parameters are a=1, b=0.5, c=0.5, Δω=0.1). The curvature around the new fixed points of the system (shown by the vertical solid and dashed lines on the left) is shown on the right for the (close to) in-phase and anti-phase patterns (solid and dashed black lines, respectively. The curvatures under the original model are shown by corresponding gray lines). Although the intentional forcing term is stabilizing the anti-phase pattern in this example, the critical point is actually shifted to the right due to the detuning, meaning that one may expect transitions at lower frequencies. The two blue diamonds indicate the value of the control parameters matching the potential shown on the left (b/a=0.5).

Putting it all together (Figure 10), the extended HKB model, including an intentional term, is thus given by

(8) $$\begin{array}{cc} \dot{\phi } & =-\frac{d}{d\phi }V\left(\phi ,\phi_{0}\right)+\sqrt{Q}\xi_{t}\\ V(\phi ,\phi_{0}) & =-\Delta \omega \;\phi -a\cos\left(\phi \right)-b\cos\left(2\phi \right)-c\cos\left(\phi -\phi_{0}\right) \end{array}$$

#### 3.3.3. Pattern Stability

**Figure 11:**
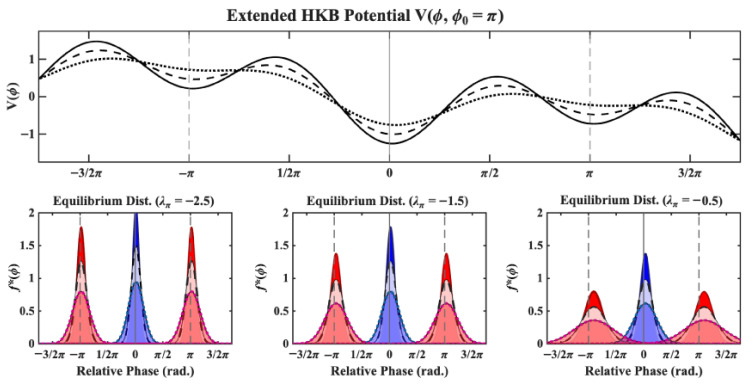
(**Top**) The extended HKB potential $V(\phi ,\phi_{0})$ plotted against ϕ (in radians) for three values of the curvature around the anti-phase pattern $\lambda_{\pi }$ (see Equation (7)) assuming intentional forcing of the anti-phase pattern ($\phi_{0}=\pi$). The solid, dashed and dotted black curves correspond to a highly stable ($\lambda_{\pi }=-2.5$), moderately stable ($\lambda_{\pi }=-1.5$) and weakly stable ($\lambda_{\pi }=-0.5$) anti-phase pattern, respectively. (**Bottom**) The equilibrium distributions for the relative phase $f^{*}\left(\phi \right)$ obtained under a quadratic expansion around the in-phase (blue curves) and anti-phase (red curves) patterns of the three potential landscapes shown at the top. The equilibrium distributions are shown for three different values of the noise magnitude term *Q*, with solid, dashed and dotted curves corresponding to Q=0.25, Q=0.75 and Q=1.25, respectively. As can be observed, the standard deviation, corresponding to the width of these equilibrium distributions, grows with the magnitude of the noise (*Q*) and shrinks with the pattern’s dynamical stability ($\lambda_{\phi }$).

To relate this model to experimentally measured quantities (i.e., the mean and SD of relative phase), we followed along the line of Schöner, Haken, and Kelso (1986) [33] by first solving for the equilibrium density of the Fokker–Planck equation associated with Equation (8) using a quadratic expansion of $V(\phi ,\phi_{0})$ around the in-phase $\phi^{*}=0$ and anti-phase patterns $\phi^{*}=\pi$. Analytical formulae for the mean and standard deviation of the equilibrium densities can then be obtained by simply applying their definitions. Assuming $\phi_{0}=\phi^{*}$ (i.e., the participant is stabilizing the pattern they are performing), the Fokker–Planck equation is given by

(9) $$\begin{array}{cc} \dot{f}\left(\phi ,t\right) & =-\frac{\partial }{\partial \phi }\left[-\frac{\partial }{\partial \phi }V_{\phi^{*}}\left(\phi \right)f\right]+\frac{Q}{2}\frac{\partial^{2}}{\partial \phi^{2}}f\\ V_{\phi^{*}}\left(\phi \right) & =\frac{-\lambda_{\phi^{*}}}{2}\phi^{2}-\Delta \omega \;\phi \end{array}$$

where $V_{\phi^{*}}\left(\phi \right)$ is the approximation of $V(\phi ,\phi_{0})$ around $\phi^{*}$ assuming $\phi_{0}=\phi^{*}$ and $\lambda_{\phi }^{*}$ is given by (7) where *c* counts negatively. Solving for the equilibrium density $f^{*}\left(\phi \right)$ yields

(10) $$\begin{array}{c} f^{*}\left(\phi \right)=N^{-1}e^{-{\left(k_{\phi^{*}}\phi \right)}^{2}+\mu \phi }\\ k_{\phi^{*}}={\left(\frac{-\lambda_{\phi^{*}}}{Q}\right)}^{1/2}\;\;\;\;\;\mu =\frac{2\Delta \omega }{Q} \end{array}$$

where N normalizes $f^{*}\left(\phi \right)$ within the interval $\left[\phi^{*}-\pi ,\phi^{*}+\pi \right]$. Figure 11 shows examples of equilibrium densities obtained under different values of $\lambda_{\pi }$ and *Q*. Hereafter, we refer to the aggregated parameters $k_{\phi^{*}}$ and μ as the $\phi^{*}$ pattern *stochastic stability coefficient* (since it aggregates both $\phi^{*}$ dynamical stability and the noise magnitude *Q*) and the *stochastic detuning coefficient* (since it aggregates detuning Δω and and the noise magnitude *Q*).

An expression for the mean and standard deviation of $f^{*}$ can be obtained by first calculating its first trigonometric moment, $M_{1}$:

(11) $$\begin{array}{cc} M_{n} & =\int_{-\pi }^{\pi }f^{*}\left(\phi \right)e^{in\phi }d\phi \\ \; & =-e^{-\nu^{2}-{\left(\gamma_{n}-i\nu \right)}^{2}}\left[\frac{\mathrm{erfi}\left(\gamma_{n}+iz^{+}\right)-\mathrm{erfi}\left(\gamma_{n}+iz^{-}\right)}{\mathrm{erfi}\left(iz^{+}\right)+\mathrm{erfi}\left(iz^{-}\right)}\right]\\ \; & \gamma_{n}=n/2k_{\phi^{*}}\;\;\;\;\;\;\nu =\mu /2k_{\phi^{*}}\\ \; & z^{+}=k_{\phi^{*}}+\nu \;\;\;\;\;z^{-}=k_{\phi^{*}}-\nu \end{array}$$

where $M_{n}$ is $f^{*}$’s trigonometric moment of order *n*, $i^{2}=-1$ is the complex variable, and erfi(z) is the complex error function. The circular mean (*m*) and standard deviation (*s*) are finally given by

(12) $$\begin{array}{cc} m & =\arg(M_{1})\\ s & =\left[-2\log(\right|M_{1}{\left|)\right]}^{1/2} \end{array}$$

**Figure 12:**
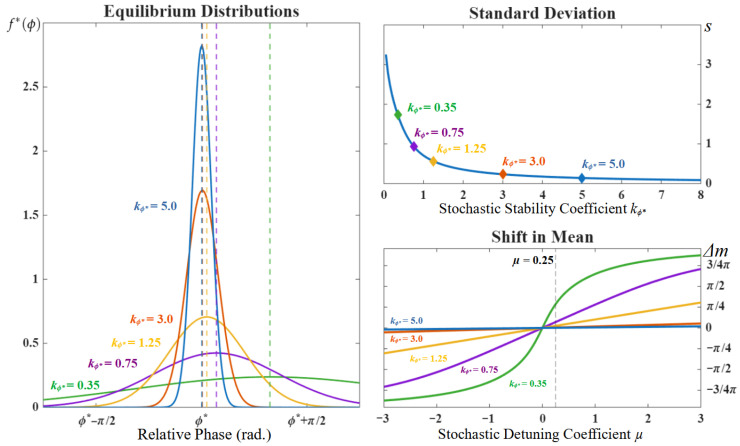
(**Left**) The equilibrium distribution of the relative phase under a pattern $\phi^{*}$ for different values of the pattern stochastic stability ($k_{\phi^{*}}={\left(-\lambda_{\phi^{*}}/Q\right)}^{1/2}$). The blue, orange, yellow, purple and green curves correspond to the equilibrium distributions obtained under μ=0.25 for $k_{\phi^{*}}=5$, $k_{\phi^{*}}=3$, $k_{\phi^{*}}=1.25$, $k_{\phi^{*}}=0.75$, and $k_{\phi^{*}}=0.35$, respectively. The vertical dashed lines denote the expectation (i.e., mean) of each distribution. (**Top right**) The standard deviation of the relative phase equilibrium density under a pattern $\phi^{*}$ as a function its stochastic stability $k_{\phi^{*}}$. The diamonds mark the values associated with the distributions shown on the left (with matching colors). (**Bottom right**) The shift in mean, (i.e., distance between the expectation of the equilibrium distribution and the pattern $\phi^{*}$) as a function of stochastic detuning (μ=2Δω/Q) for different values of the pattern stochastic stability ($k_{\phi^{*}}$). The color of each curve indicates the value of $k_{\phi^{*}}$ matching the equilibrium distributions shown on the left. The vertical dashed line indicates the value μ=0.25.

Figure 12 shows how the equilibrium density, the stochastic stability coefficient $k_{\phi^{*}}$ and the stochastic detuning parameter μ relate to the experimentally measured mean and standard deviation.

#### 3.3.4. Intention and Fluctuations

Having derived a relationship between the mean and standard deviation of the relative phase with the parameters of an extended HKB model that includes a term representing a participant’s intention to stabilize a given coordination pattern, we now come back to our core question: which parameters in this model does the participant’s intention act upon? In particular, is the large standard deviation we observed as participants held on to the anti-phase pattern at super-critical frequencies explained solely by the continued decrease in that pattern’s dynamical stability (i.e., $\lambda_{\phi }$ in Equation (7), due to to the continued increase in pacing frequency) or does their intention also increase the stochasticity of the dynamics (i.e., *Q* in Equation (8), reflecting the magnitude of the ‘noise’)?

The answer to this question depends on the value of the stochastic stability coefficient measured right before the spontaneous transition in the Let-go Anti-phase trial (the critical $k_{\phi^{*}}$). Note that this critical value of $k_{\phi^{*}}$ indicates the parameter regime under which a spontaneous transition is likely to occur in the absence of intention (i.e., when participants let go, where we assume c=0 and thus the dynamics correspond to the original model). This critical $k_{\phi^{*}}$ value furnishes a threshold against which we can compare the estimate of $k\phi^{*}$ obtained when holding onto anti-phase at super-critical frequencies (the super-critical $k_{\phi^{*}}$). Should the super-critical $k_{\phi^{*}}$ (hold-on) be larger than the critical $k_{\phi^{*}}$ (let-go), the anti-phase pattern we observed as the participant was holding on exists in a parameter regime that matches the original HKB model. In this first scenario, the effect of the participant’s intention is to compensate for the loss of dynamical stability of the anti-phase pattern due to the increased pacing demand. This explains how the transition can be prevented or delayed while maintaining a constant level of fluctuations (i.e., the parameter *Q*) in the system. Conversely, if the super-critical $k_{\phi^{*}}$ (hold-on) is smaller than the critical $k_{\phi^{*}}$ (let-go), the observed anti-phase pattern exists in a parameter regime wherein the original HKB model predicts that a transition is extremely likely. Since we do not observe a transition to in-phase here, this is evidence that the anti-phase pattern is in fact the only stable state when the participant is holding on. In other words, the intentional strength parameter, *c*, must be of sufficient magnitude that the intentional potential now drives the dynamics. Since the stochastic stability coefficient necessarily grows with *c* (i.e., per Equation (7)) and shrinks with the magnitude of fluctuations *Q*, the relatively small value of the super-critical $k_{\phi^{*}}$ despite a large *c* necessitates an increase in *Q* accordingly.

**Figure 13:**
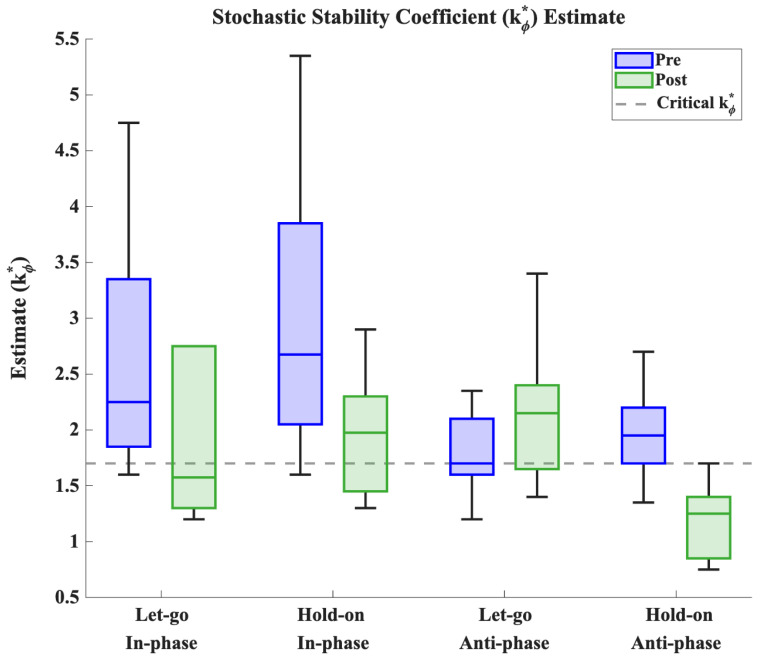
Estimates of the stability coefficient $k_{\phi^{*}}$ obtained from the frequency plateau immediately before (pre, in blue) and after (post, in green) the transition observed in the Let-go Anti-phase trial in all trials. The critical $k_{\phi^{*}}$ (dashed gray line) determines the value below which the anti-phase pattern is stochastically unstable under the original HKB potential.

Since the standard deviation is a monotonously decreasing function of the stochastic stability coefficient, as shown in the top right of Figure 12, the fact that the standard deviation is large when participants hold on at super-critical frequencies points to the second scenario detailed above. This was confirmed by estimating, for each participant and for all trials, the value of the stability coefficient in the frequency plateaus surrounding the phase transition in the Let-go Anti-phase trial (see Figure 13). The stability estimate was obtained by matching the standard deviation measured in the two plateaus before and after the phase transition to a value of $k_{\phi^{*}}$ using the graph shown in the top right of Figure 12. Comparing the estimates obtained pre-transition in the Let-go Anti-phase trial ($k_{\phi^{*}}=1.77\pm 0.37$ *SD*), which serves as our critical value, to the estimates obtained post-transition in the Hold-on Anti-phase trial ($k_{\phi^{*}}=1.20\pm 0.34$ *SD*), we find that the latter is indeed significantly smaller [t(9)=3.98;d=1.52;p=0.003], indicating that the relative phase dynamics is now largely driven by the intentional term (i.e., *c* is large) and that the magnitude of the noise *Q* must have grown accordingly. Within the confines of our proposed model, this result indicates that the participants’ intention to hold on to the anti-phase pattern at super-critical frequencies is accompanied by an increase in stochasticity.

## 4. Discussion

Here we used the bimanual coordination paradigm as a window to investigate the relationship between intention and fluctuations. In line with previous research [25], instructing participants to hold on to an intrinsically stable pattern does not appear to affect the dynamics of bimanual coordination. On the other hand, at subjectively challenging cycling frequencies (i.e., frequencies at and beyond an individual’s critical frequency), the intention to hold on can stabilize an otherwise unstable coordination pattern while simultaneously increasing pattern fluctuations.

To accommodate these empirical findings, we showed how the order parameter dynamics of an extended HKB model can be transformed to accommodate participants’ intention to hold on. Although different modeling choices might lead to different conclusions, the HKB model is one of the few models of human behavior that makes experimentally verifiable predictions across many domains and scales of human activity (e.g., social dynamics, economics, brain dynamics, learning, gait transitions, human–machine coordination, etc., see [35]). HKB’s non-linear dynamics are deemed to arise as a result of emergent, self-organizing processes. The difference in stability between in-phase and anti-phase coordination patterns, their status as the sole intrinsically stable coordinative states, and the transition between them (and many other predictions [9,11,15]) follow from the simple (again empirically-based) assumption that the dynamics of bimanual coordination can be captured by two (non-linearly) coupled non-linear oscillators. In short, our model-based inferences rest upon a foundation of empirical support backed up by theoretical modeling.

To echo George Miller, insofar as intention is a fundamental aspect of human behavior it is also highly elusive. One thing we can however say about intention that may bring things to bear is that it does not operate in a vacuum. The coordination patterns that underlie intentional behavior have a dynamics of their own which delineate and constrain what one can be intentional about [11,15]. As such, to the extent that the original HKB model captures the intrinsic dynamics of coordinated oscillations (and there is good reason to believe that it does), what is demonstrated here is that the intent to hold on acts in such a way that it both stabilizes (large *c*) *and* destabilizes (large *Q*) unstable coordination patterns. While we cannot yet make claims about what intention actually is, understanding intrinsic dynamics and measuring the way they transform when intention is involved allows us to make claims about what intention *actually does*.

Of course, the work presented here only scratches the surface. Further study, involving larger sample sizes, trial repetitions, and lengthier trials would help to more precisely quantify the way intention may increase the level of noise in the collective dynamics. Nevertheless, it is intuitively and theoretically surprising, under traditional models of motor control, that coordination can become more variable when a person tries to stabilize a specific pattern. Such models assert that the brain acts more or less like a computer, storing a number of specific motor programs and selecting from among them on the basis of their utility with respect to a given goal (see, e.g., active inference [38,39], integrated models of cognition based on production systems [40,41] or forward–inverse models of sensorimotor control [42,43,44,45]). The idea of a motor program was introduced to explain how typical actions (such as reaching for a glass of water) can be achieved even though they involve the coordination of a tremendous number of degrees of freedom. While the details vary from one theory to another depending on the phenomena they aim to describe or the problems they were designed to solve, all are based on a cybernetic conception of cognition which likens cognitive function to a regulator, under which mental acts arise from the impetus provided by goals—the most fundamental goal being the maintenance of homeostasis. Once a motor program has been selected as a solution to a specific goal, it prescribes an ideal trajectory for the motor system. The actual trajectory will, however, need to be monitored and updated continuously, since biological systems are open to interactions with the environment and random, external perturbations are inevitable. ‘External’ is the keyword here, as the biological system does not fluctuate by itself within typical cybernetic frameworks. Rather, the environment to which it is coupled does. Fluctuations in this picture are understood as errors and intention conceived as the manifestation of a regulatory process aimed at suppressing and correcting for those errors to achieve a goal.

Following this line of thinking, an intention to stabilize a target pattern (in the model, this corresponds to the case where c>0) should engage this regulatory process and result in suppression of deviations (i.e., ‘error’) from the intended coordination pattern—no matter the degree to which the target pattern is intrinsically stable. However, that is not what we observed in the current study. Not only does the intention to stabilize a given pattern have no significant effect on the dynamics when this pattern is intrinsically stable, it also increases the magnitude of the fluctuations when the intended pattern is unstable. In other words, clean separation into a deterministic motor program perturbed by a noisy environment is not tenable as an account of behavior. As evidenced by the current results, intentionality must come with a deterministic “stabilizing” component to explain how the participants are able to intentionally prevent or delay the transition from anti-phase to in-phase (or phase wandering). At the same time, this deterministic stability must be paired with growing stochasticity to account for the fact that the stability index $k_{\phi }$ is lower when holding on at super-critical frequencies.

The peculiar relationship between fluctuations and intentionality exposed by such results is better handled by an alternative perspective provided by coordination dynamics (CD). Rather than invoking internal goals or motor programs to handle dimensionality reduction, CD recognizes that interacting components of a complex system may produce new qualitative features at large scales. In other words, the system self-organizes such that it becomes possible to describe the collective behavior of its many components using a few quantities known as order parameters. In the context of bimanual coordination, the order parameter corresponds to the relative phase between the two hands. Furthermore, order parameters not only describe collective behavior of the individual components; order parameters enslave the behavior of the parts, resulting in enormous information compression. Order parameters are strongly determined by so-called boundary conditions, which may generally be divided into structural constraints (e.g., the apparatus restricting the finger’s movement about the metacarpophalangeal joint in the present experiment) and energizing conditions (e.g., the metronome driving the fingers to oscillate at increasingly high frequencies and the instructions provided by the experimenter), with the latter being represented mathematically as control parameters. It is these control parameters that, when changed, drive the system close to its instability points and allow for the emergence of a new structure or behavior (e.g., the transition from anti-phase to in-phase).

The apparent intentionality of the system emerges through the interplay of dynamical constraints of the organism and environment, namely the order parameters and control parameters, which often present themselves in the form of a gradient. A reformulation of the second law of thermodynamics states that a system under stress (by an externally applied non-equilibrium gradient) will use any avenue available to return to equilibrium [46]. This means that in the absence of intention, intrinsic dynamics correspond to those behaviors that guide the system toward greater stability. However, when intention is at odds with intrinsic dynamics, the effect must be to disrupt this flow to stability, keeping the system far from equilibrium. From this theoretical standpoint, the will to resist a spontaneous return to stability should be marked by an increase in fluctuations. These theoretical predictions perfectly align with our experimental findings that (1) holding on does not further stabilize the in-phase pattern, and (2) holding on to the anti-phase pattern at subjectively challenging cycling frequencies can stabilize an otherwise unstable coordination pattern while simultaneously increasing fluctuations in that pattern.

More broadly, this approach offers a way to handle intentionality without abandoning thermodynamic principles (i.e., by invoking a homunculus-like internal operator). Biological systems are characterized by their directed, purposeful, and functional behavior, but terms like ‘intention’ and ‘purposeful’ tend to lead one towards spooky territory well outside the purview of standard physics. By reframing intentionality in terms of the dynamics of a complex, non-linear system operating far from equilibrium (e.g., humans), ‘meaning’, ‘purpose’ or ‘intent’ simply reflect self-organization through an interaction of constraints. Since order parameters emerge through the dissipation of externally applied non-equilibrium gradients [47,48], they relate to the environmental conditions (described by control parameters) to which the system is responding, and order parameters are functional in that they are directed toward reducing or destroying the externally applied gradients. Directedness toward some functional end is a key ingredient of intention.

Crucially for the present discussion, self-organization also offers a completely different perspective on the relationship between fluctuations and intention. This is because order parameters describe the slow modes of the dynamics while the fast modes they enslave are adiabatically eliminated and taken as small perturbations [49]. In other words, fluctuations in the order parameters of a self-organizing system do not come solely from the outside but reflect the fast modes of the dynamical matrix from which they emerge. As such, they are as much internal as they are external to the system under study. For self-organizing systems, fluctuations are seldom errors but rather are an intrinsic and integral part of coordination dynamics.

### The Functional and Fractal Nature of Intentional Fluctuations

Adopting a conventional motor control theoretic stance paints a picture of intentional behavior as a drift-diffusion like process, wherein the dynamics separate into a deterministic drift prescribed by an internal motor program and a stochastic diffusion induced by external perturbations to the program’s enactment. Random forces embedded in the latter make the system deviate from its ideal trajectory and are therefore taken as error that must be suppressed for the system to reliably achieve its goals. From an experimental point of view, one should thus observe an inverse relationship between intentions and fluctuations, and fluctuations should exhibit a Gaussian structure. Neither of these predictions are supported by empirical study. Typically, the temporal structure of fluctuations which biological systems exhibit does not conform to that of thermal noise. Taking bimanual coordination as an example, studies have shown that the spectrum of the relative phase fluctuations is characterized by an inverse power law relationship between frequency and intensity rather than the constant power spectral density of thermal noise (see e.g., [50]). The ubiquity of $1/f^{\alpha }$-noise in biological systems, and in particular human behavior (see [51,52] and references therein), is thought to be the fingerprint of an “interaction dominant system” [53,54], whose global behavior is driven by self-organization and emergence at the level of the whole rather than by the integrated activities of its constituent parts or specialized “modules”.

Conversely, when treating intentional states in terms of order parameters, the separability of the dynamics into drift and diffusion aspects does not follow from internal and external processes, for the existence of the deterministic drift prescribed by the order parameters (the slow modes) is conditioned on the existence of a stochastic diffusion reflecting the part of the dynamic they enslave (the fast modes). In other words, intentions and fluctuations are deeply intertwined, and one cannot exist without the other. From an experimental point of view, one should thus be open to a broader range of interrelationships including, as we have shown here, an increase in fluctuations (larger *Q*) when the dynamics are subjected to intention (c>0) depending on the boundary conditions (in this case beyond the critical/switching frequency).

Furthermore, fluctuations hold functional significance for perception [55,56,57], action [58,59], learning [60,61] and decision-making [62]. Far from being dismissible noise, fluctuations are essential functional elements in the complex non-linear dynamics of biological systems. If intentional states are treated as order parameters of a biological system, fluctuations must then play a key role: first, because order parameters grow out from fluctuations in the dynamical skeleton consisting of the slow modes of the dynamics [63], and second, because stochastic diffusion is the mechanism by which the system can probe the stability of its attractor state and flexibly transition between states [9,33,49]. In other words, fluctuations are a necessary requirement both for a complex system to exhibit organized, intentional behavior (i.e., for order parameters to emerge) as well as to adapt behavior to the boundary conditions under which it unfolds (select among attractor states).

## 5. Conclusions

Here we conducted a simple experiment to illustrate and explore the effects of intention on coordinative stability. In line with previous findings, we observed that holding on to a coordinative pattern when it would be easier to let go preserves the overall pattern but renders that pattern increasingly variable. Theoretically, we show that such increasing variability can be attributed to an increase in the stochasticity of the order parameter dynamics prescribed by an extended HKB model. Intentional states and stochastic fluctuations are conventionally understood as opposing forces. Mental phenomena are thought to arise from the drive to achieve goals, and staying alive is a fundamental goal of any living system. As such, fluctuations are assumed to arise from external noise pushing the system towards thermal equilibrium, while internally generated intentions maintain the system in a highly ordered state necessary for life. Here, we offer a radically different picture in which fluctuations are a necessary complement to mental states. Characterizing in more detail how the relationship between intentional states and fluctuations plays out, both from theoretical and empirical perspectives, may further clarify the nature of Schrödinger’s “I”. In closing, we note that there has been a long history concerning the brain structures involved in the generation of voluntary movements. There have even been models of how the strength of intention can influence elementary processes in cortical areas such as the supplementary motor area of the cerebral cortex (see, e.g., [64], see also [65]). While certainly of interest, the focus of the present work has been to establish—by the usual means of experiment and theoretical modeling—a fundamental (quantitative) connection between intention and fluctuations in the elementary laws of coordination.

## Figures and Tables

**Table 1:** The average mean and standard deviation (*M*(*SD*), in rad) of the relative phase for the participants who exhibited a phase transition in the Let-go Anti-phase trials as a function of starting pattern (in-phase or anti-phase), intention (Let-go or Hold-on) and metronome frequency (low, medium and high). Low metronome frequency corresponds to the 1.00 Hz to 1.50 Hz plateaus, medium to the 1.75 Hz to 2.25 Hz plateaus and high to the 2.50 Hz to 3.00 Hz plateaus.

		Condition			
		*In-phase*		*Anti-phase*	
		Let-go	Hold-on	Let-go	Hold-on
**Metronome Frequency**	*Low*	0.06 (0.31)	0.07 (0.29)	2.99 (0.57)	3.26 (0.56)
	*Medium*	−0.01 (0.39)	0.00 (0.40)	0.93 (0.54)	3.04 (0.54)
	*High*	−0.05 (0.55)	0.04 (0.53)	0.63 (0.60)	2.33 (0.92)

## Data Availability

The anonymized data are available from the corresponding author on reasonable request.
